# Genepanel.iobio - an easy to use web tool for generating disease- and phenotype-associated gene lists

**DOI:** 10.1186/s12920-019-0641-1

**Published:** 2019-12-11

**Authors:** Aditya Ekawade, Matt Velinder, Alistair Ward, Tonya DiSera, Chase Miller, Yi Qiao, Gabor Marth

**Affiliations:** USTAR Center for Genetic Discovery, Department of Human Genetics, University of Utah School of Medicine, Salt Lake City, UT USA

## Abstract

When ordering genetic testing or triaging candidate variants in exome and genome sequencing studies, it is critical to generate and test a comprehensive list of candidate genes that succinctly describe the complete and objective phenotypic features of disease. Significant efforts have been made to curate gene:disease associations both in academic research and commercial genetic testing laboratory settings. However, many of these valuable resources exist as islands and must be used independently, generating static, single-resource gene:disease association lists. Here we describe *genepanel.iobio* (https://genepanel.iobio.io) an easy to use, free and open-source web tool for generating disease- and phenotype-associated gene lists from multiple gene:disease association resources, including the NCBI Genetic Testing Registry (GTR), Phenolyzer, and the Human Phenotype Ontology (HPO). We demonstrate the utility of *genepanel.iobio* by applying it to complex, rare and undiagnosed disease cases that had reached a diagnostic conclusion. We find that *genepanel.iobio* is able to correctly prioritize the gene containing the diagnostic variant in roughly half of these challenging cases. Importantly, each component resource contributed diagnostic value, showing the benefits of this aggregate approach. We expect *genepanel.iobio* will improve the ease and diagnostic value of generating gene:disease association lists for genetic test ordering and whole genome or exome sequencing variant prioritization.

## Background

A tremendous amount of genetic and biomedical knowledge has been deposited in both curated and computationally-derived gene:disease association database resources such as NCBI’s Genetic Testing Registry (GTR) [6], Phenolyzer [8], and the Human Phenotype Ontology (HPO) [5]. However, each resource has its own format, data structure and output, making it difficult for a genetics professional to navigate between resources; and especially difficult to merge the outputs of these resources into a concise, non-redundant list that encompasses the phenotypic features of disease. As such, there is a strong need in the genetics and genomics community for a tool capable of consolidating and harmonizing these gene:disease association resources and providing genetics professionals with a single prioritized disease-candidate gene list.

## Implementation

**Fig. 1:**
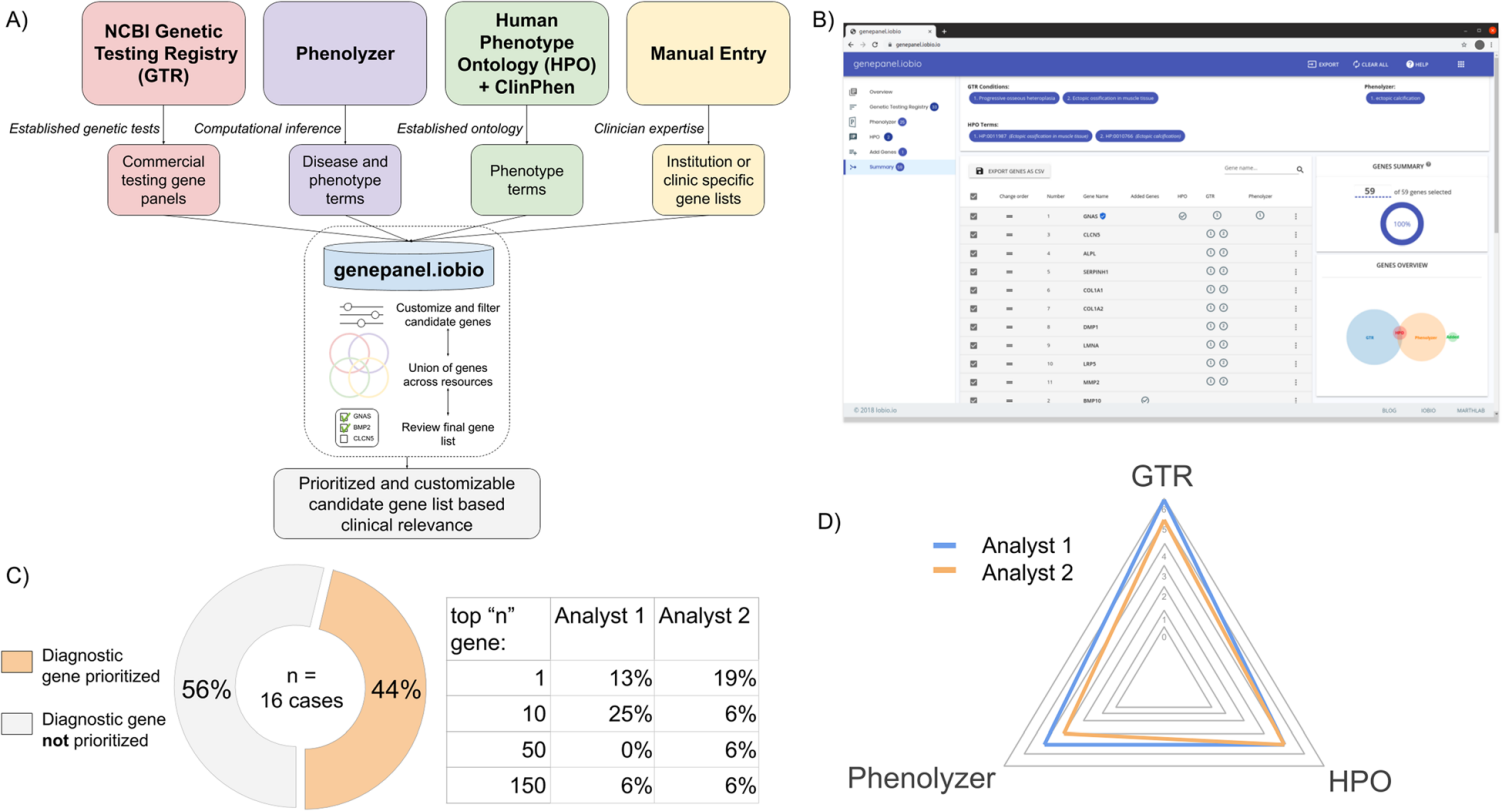
An overview of genepanel.iobio applied to clinical genetics cases **a** a diagrammatic view of the resources genepanel.iobio utilizes and how genepanel.iobio presents a prioritized and unioned set of candidate genes **b** a screenshot view of the summary page of a genepanel.iobio analysis, showing the ability to modify and customize the final gene list and export the results **c** two independent analysts correctly prioritize the diagnostic gene using genepanel.iobio in 7 of the 16 clinical cases (44%) tested and where the diagnostic gene was ranked in the final gene list by each analyst **d** radar-plot summary of which resources correctly prioritized the diagnostic gene, allowing for multiple resources to identify the same gene

Our primary goal in developing *genepanel.iobio* was to make it as universally accessible as possible, ensuring clinicians and genetics professionals without prior computational expertise could adopt it into their clinical workflows. This goal lent itself towards a web-browser application capable of being run on any computer, regardless of operating system or hardware specifications and reflects our goals for the *iobio* platform more generally [4, 7]. Accordingly, we built *genepanel.iobio* to fully utilize the modern progressive component-based Javascript framework, Vue. Vue allows us to write, reuse and share components for creating a platform-independent application that runs entirely in a web browser. Within this framework we can fetch data from various application programming interfaces (APIs) such as the NCBI’s Genetic Testing Registry [6] (Fig. 1a). Querying data via an API ensures the *genepanel.iobio* user is retrieving the most up-to-date information available. For tools without an available API (Phenolyzer [8] and ClinPhen [2]) we developed backend services, written in Node.js, that create a wrapper around the original command-line tools. We optimized these backend services numerous ways, for example, by caching Phenolyzer’s output in DynamoDB (a NoSQL database), allowing for near instantaneous results of previously searched terms. The application is hosted on AWS S3 and utilizes AWS Cloudfront services. This provides a fast content delivery network which is massively scaled and globally distributed with superior performance and stability. The application is also configured with HTTPS to ensure the user has secure end-to-end connections to the origin servers. Numerous other design considerations were implemented based on clinician feedback, including: type-ahead prediction for terms; changes to the user-interface; advanced filtering for each resource; adding additional resources such as HPO terms; export features; and displaying pertinent gene information for each gene. On the whole, this approach has allowed us to harmonize disparate data sources, structures and programs into a single easy-to-use, broadly adoptable application. *Genepanel.iobio* was developed and tested on the Google Chrome browser. We recommend using the most up-to-date version of Chrome to ensure stability and performance. Our code is free, open-source and available at https://github.com/iobio/genepanel.iobio.

## Results

Within a typical *genepanel.iobio* usage, a user provides relevant terms to each resource tool. For instance, starting with the NCBI Genetic Testing Registry input step, the user will enter one or more presumed genetic disorder terms, selecting the term from the typeahead drop down. In the Phenolyzer input step, the user searches and then selects one or more disease-relevant phenotype terms. In the HPO input step the user can either directly input HPO terms or copy/paste a clinical note and allow the built-in ClinPhen [2] tool to identify and rank relevant HPO terms from the clinical note (Fig. 1a). These input steps can be performed independently of one another, allowing the user to utilize some or all of the available resources. Within each input step the user can apply advanced resource-specific filters to further customize and refine the genes generated by that resource. For example, in the GTR step, the user can filter on commercial testing providers and/or disease modes of inheritance, etc. Following data input *genepanel.iobio* presents the user with a summary page of the prioritized union of genes across all resources where data has been inputted. This final summary page also allows the user to further refine and modify the gene list and export a final list of genes to a text file, comma-separated file or copy them to the system clipboard (Fig. 1b).

Following development, we tested the efficacy of *genepanel.iobio* to correctly prioritize diagnostic variants in a clinical genetics setting. We chose an ambitious test setting, selecting cases for which diagnostic conclusions had been reached previously at the Penelope Undiagnosed and Rare Disease Program at the University of Utah. The rate of diagnosis in this setting remains between 35 and 45%, often complicated by complex clinical presentations and ambiguous or blended phenotypes [1, 3]. Two analysts, blinded to the causal variants, independently analyzed 16 previously diagnosed clinical cases to see if *genepanel.iobio* could correctly prioritize the gene containing the diagnostic variant within the final gene list. These analysts, with no speciality or expert knowledge of the genetic disorders or the genes associated with them, were able to correctly prioritize the diagnostic gene in their final gene list in 7 of the 16 clinical cases (44%). The total number of genes in the final gene list were typically around 150 genes, a number we deemed reasonable for gene panel testing and genetic sequencing variant review workflows. The diagnostic gene was the number one ranked gene in the final gene list for 2 (analyst 1) and 3 (analyst 2) of the clinical cases. Additionally, for over one third of cases (44% analyst 1, 38% analyst 2), the diagnostic gene was in the top 50 genes of the final gene list (Fig. 1c). Importantly, each resource contributed to the analysts’ ability to correctly prioritize the diagnostic gene (Fig. 1d). For the cases where *genepanel.iobio* failed to correctly prioritize the diagnostic gene, we attribute a large degree of these failures to the lack of objective findings in the phenotype descriptions (Additional file 1: Table S2). These challenging cases were largely described in less descriptive terms such as developmental delay, growth delay, hypotonia and macrocephaly. Additional metrics about the gene lists generated and phenotype descriptions given to the analysts can be found in Additional file 1: Table S1 and S2.

These results demonstrate the benefit of *genepanel.iobio* and its aggregate approach of using multiple gene:disease association resources. We are actively developing and maintaining *genepanel.iobio* and will be incorporating new features and resources in the future.

## Conclusion

Genomic medicine has greatly benefited from the increasing wealth of knowledge in gene:disease association databases and resources. However, it remains difficult to harmonize results across multiple such resources. To address this difficulty we developed *genepanel.iobio*, a free, open-source, platform independent web application capable of generating a comprehensive list of genes associated with a user-provided set of suspected disorders and phenotypes. We demonstrate the utility of *genepanel.iobio* in a clinical genetics setting by its ability to generate a gene list containing a reasonable number of genes that described the clinical phenotype and most importantly contained the diagnostic gene. We anticipate adoption of *genepanel.iobio* into clinical genetics workflows will improve the diagnostic value of genetic test ordering as well as variant/gene prioritization in genetic sequencing studies.

## Availability and requirements

Project name: genepanel.iobio

Project home page: https://genepanel.iobio.io and https://github.com/iobio/genepanel.iobio

Operating system(s): Platform independent

Programming language: Vue, Javascript

Other requirements: Chrome browser version 76 or greater

License: MIT-license

Any restrictions to use by non-academics: none

## Additional file

**Additional file 1: Table S1.** Summary of the diagnostic analysis for 16 previously diagnosed clinical cases, **Table S2.** Phenotype descriptions that were given to each analyst, whether each analyst was able to correctly identify the diagnostic gene using genepanel.iobio and a likely rationale for the analysts’.

## Data Availability

https://genepanel.iobio.io and https://github.com/iobio/genepanel.iobio

## Abbreviations

API: Application programming interface
GTR: NCBI Genetic Testing Registry
HPO: Human Phenotype Ontology
